# Evaluation of the neuroprotective effect of taurine in Alzheimer’s disease using functional molecular imaging

**DOI:** 10.1038/s41598-020-72755-4

**Published:** 2020-09-23

**Authors:** Se Jong Oh, Hae-June Lee, Ye Ji Jeong, Kyung Rok Nam, Kyung Jun Kang, Sang Jin Han, Kyo Chul Lee, Yong Jin Lee, Jae Yong Choi

**Affiliations:** 1 Division of Applied RI, Korea Institute of Radiological and Medical Sciences, 75 Nowon-ro, Nowon-gu, Seoul, 01812 Korea; 2 Division of Radiation Effects, Korea Institute of Radiological and Medical Sciences, Seoul, Korea

**Keywords:** Neurodegenerative diseases, Brain imaging, Radionuclide imaging, Drug therapy

## Abstract

Alzheimer’s disease (AD) is a chronic neurodegenerative disorder and the leading cause of dementia, but therapeutic treatment options are limited. Taurine has been reported to have neuroprotective properties against dementia, including AD. The present study aimed to investigate the treatment effect of taurine in AD mice by functional molecular imaging. To elucidate glutamate alterations by taurine, taurine was administered to 5xFAD transgenic mice from 2 months of age, known to apear amyloid deposition. Then, we performed glutamate positron emission tomography (PET) imaging studies for three groups (wild-type, AD, and taurine-treated AD, n = 5 in each group). As a result, brain uptake in the taurine-treated AD group was 31–40% higher than that in the AD group (cortex: 40%,* p* < 0.05; striatum: 32%,* p* < 0.01; hippocampus: 36%,* p* < 0.01; thalamus: 31%, *p* > 0.05) and 3–14% lower than that in the WT group (cortex: 10%,* p* > 0.05; striatum: 15%,* p* > 0.05; hippocampus: 14%,* p* > 0.05; thalamus: 3%, *p* > 0.05). However, we did not observe differences in Aβ pathology between the taurine-treated AD and AD groups in immunohistochemistry experiments. Our results reveal that although taurine treatment did not completely recover the glutamate system, it significantly increased metabolic glutamate receptor type 5 brain uptake. Therefore, taurine has therapeutic potential against AD.

## Introduction

Alzheimer’s disease (AD) is the most common cause of dementia (60–70%) in elderly individuals and leads to problems including memory loss, cognitive deficits, and intellectual disabilities^1^. Many studies have reported the deposition of senile plaques and neurofibrillary tangles as key pathological features in AD^2^. The global prevalence of AD is growing rapidly, highlighting the importance of early intervention, which may reduce the cost of medical support and improve patients’ quality of life^3^. According to a previous study, diagnosing people with mild cognitive impairment before dementia can save a total of 7–7.9 trillion dollars in the US^4^. AD significantly deteriorates patients’ quality of life, and a recent study reported that AD is closely related to circadian rhythm sleep disorders^5^. Therefore, extensive effort has been dedicated to the development of new drugs that reduce the amyloid burden and decelerate disease progression; however, effective therapeutic treatments have not yet been established.

Currently, two types of therapeutic drugs for AD have been approved by the US Food and Drug Administration (FDA), and these drugs exert their function via two mechanisms. Cholinesterase inhibitors (Donepezil (Pfizer, New York USA), rivastigmine (Novartis, Basel, Switzerland) and galantamine (Janssen, Beerse, Belgium)) increase the level of synaptic acetylcholine in the central nervous system (CNS), and additional studies on several cholinesterase inhibitors are ongoing^6–8^. N-methyl-D-aspartate (NMDA) receptor antagonists [i.e., memantine (Namenda, Forest Pharmaceuticals Inc, Missouri, USA)] slows the progression of AD by inhibiting glutamate excitotoxicity^9^. A recent study reported that NMDA receptor antagonists block NMDA-related ion channels and consequently reduce the influx of calcium ions into neurons^10^. Although these drugs are particularly effective in maintaining cognitive function, their disease-modifying efficacy remains controversial^11^. Therefore, developing therapeutic treatments for AD is essential.

Taurine, 2-aminoethanesulfonic acid, is the second most abundant endogenous amino acid after glutamate in the CNS^12^. The chemical plays multiple roles in the body, including thermoregulation, stabilization of protein folding, anti-inflammation, antioxidation, osmoregulation, calcium homeostasis and CNS development^13–15^. Previous reports in the literature have revealed a lack of taurine in the brains of AD patients^16^. Arai et al.^17^ reported that postmortem brain tissues of AD patients have low concentrations of taurine in the temporal cortex compared with control patients’ brain tissues. Multiple lines of evidence suggest taurine as a therapeutic agent for AD. Recently, taurine was reported to help improve cognitive function and protect against neuropathology in an animal model of AD^18^. Jakaria et al.^19^ reported that taurine displayed therapeutic potential against neurological disorders, including AD. Santa-Maria et al.^20^ reported that taurine binds to Aβ plaques with weak antifibrillogenic effects. In addition, intravenously administered taurine prevents Aβ neurotoxicity and cognitive impairment^21^. To date, no reports of the possible side effects of taurine have been documented, and due to its nontoxic properties in the body, taurine has been used in foods^22–24^.

To date, no studies have evaluated the effects of taurine on AD via functional molecular imaging. Therefore, the aim of the present study was to investigate the effect of taurine supplementation in AD mice by glutamate positron emission tomography (PET).

## Results

### PET images

The mean PET images (30–60 min) are shown in Fig. 1. By visual inspection, the AD group showed significantly lower striatal and hippocampal uptake than the wild-type (WT) group. The AD_Taurine_ group showed relatively higher uptake than the AD group. The radioactivity of the olfactory bulb, which was due to spillover from the harderian gland, was also detected in the AD and AD_Taurine_ groups.

**Figure 1:**
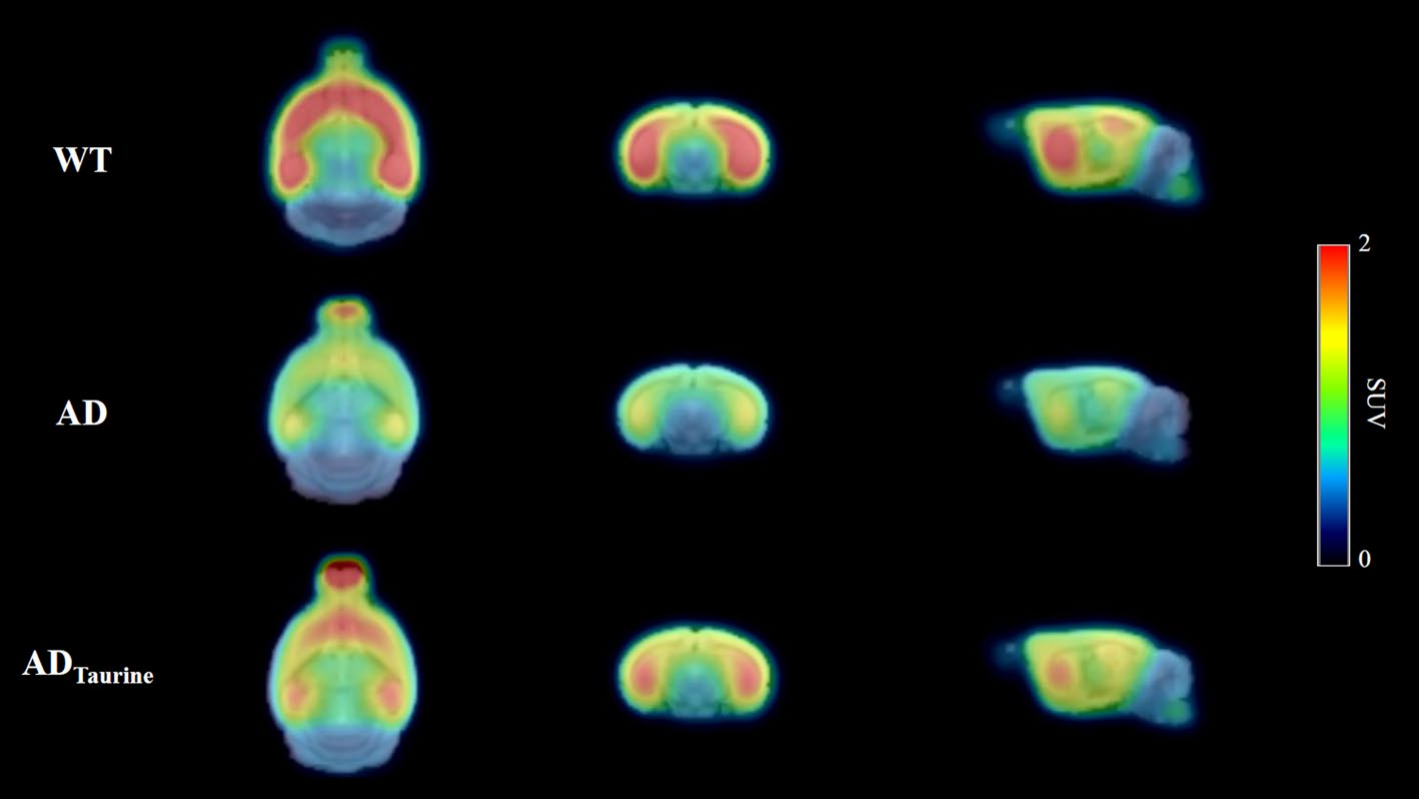
Mean ^18^F-FPEB PET images (n = 5 for each group) between 30 and 60 min after injection in the three groups. All mean PET images were created using PMOD software (version 3.4). The AD group showed dramatically lower uptake than the WT group, and the AD_Taurine_ group exhibited relatively higher uptake than the AD group. Images are shown scaled to the SUV.

### Time-activity curves (TACs)

**Figure 2:**
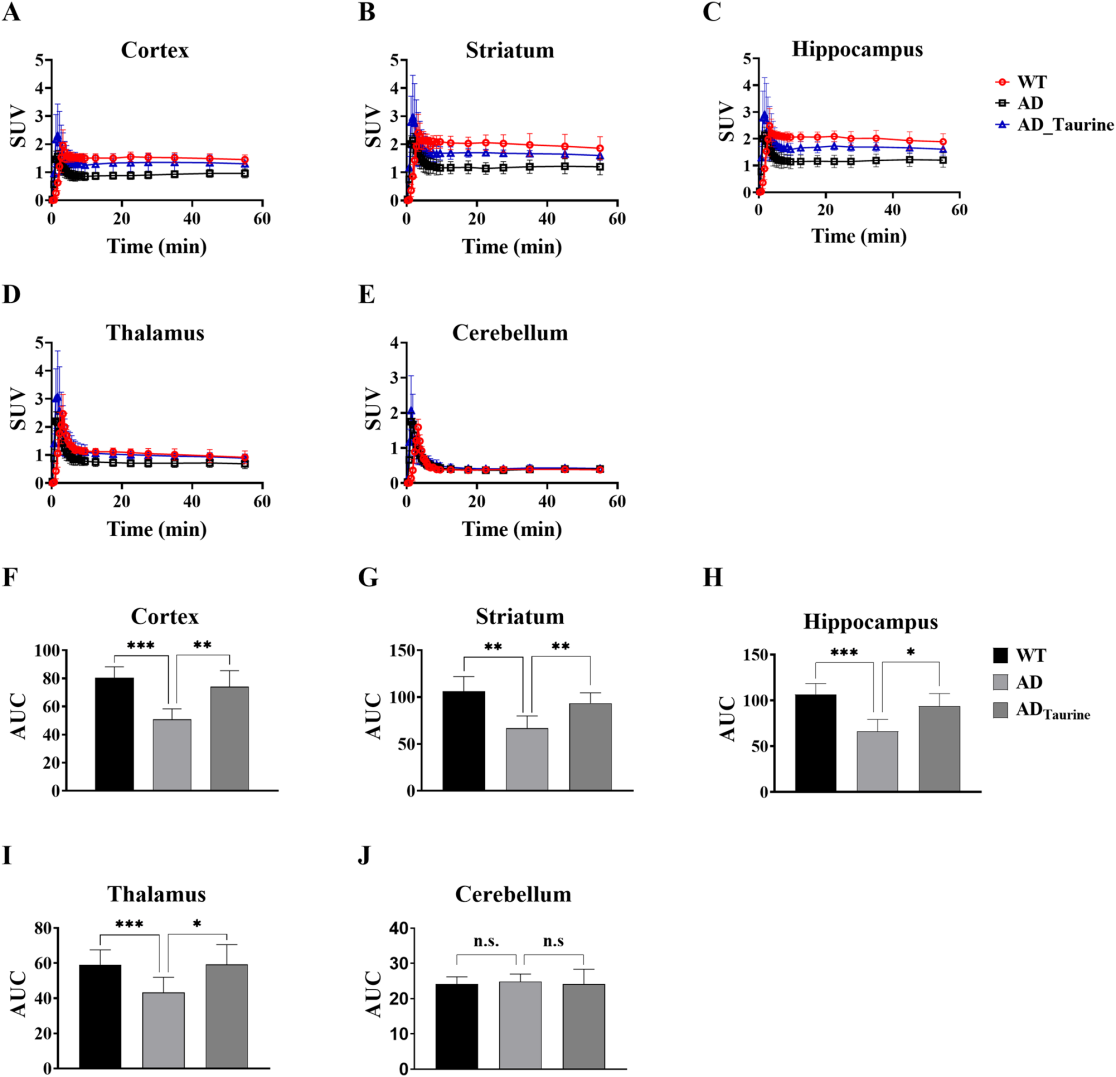
Time-activity curves of the cortex, striatum, hippocampus, thalamus and cerebellum regions (**A**–**E**). Brain uptake in the taurine-treated AD group was higher than that in the AD group but lower than that in the WT group. AUC values for the target regions (**F**–**J**). AUC values in the AD group were lower than the corresponding values in the WT group, but the AD_Taurine_ group showed higher AUC values than the AD group. Data represent the mean values ± SD (n = 5). *p < 0.05, **p < 0.01, ***p < 0.001, n.s. = statistically nonsignificant difference. Mean values were calculated and statistical analysis was performed using Prism software (version 8).

Figure 2A–E shows the regional TACs for all groups. After approximately 10 min, the radioactivity of all target regions indicated lower uptake in the AD group than in the WT group. However, the AD_Taurine_ group showed relatively higher uptake than the AD group. The uptake at 50 min post-injection (p.i.) for the target regions in the AD group was 25–36% lower than that in the WT group (Table 1). The radioactivity of the target regions was 31–40% higher in the AD_Taurine_ group than in the AD group. The radioactivity in the cerebellum, as the reference region, was similar among all groups.

**Table 1:** Comparison of SUVs for the target regions at 50 min p.i.

Group	Volumes of interest			
	Cortex	Striatum	Hippocampus	Thalamus
WT	1.49 ± 0.17	1.93 ± 0.42	1.93 ± 0.33	0.97 ± 0.23
AD	0.96 ± 0.14	1.22 ± 0.27	1.22 ± 0.25	0.72 ± 0.40
AD_Taurine_	1.34 ± 0.28*	1.65 ± 0.16**	1.66 ± 0.21**	0.94 ± 0.15

Brain uptake in the taurine-treated AD group was higher than that in the AD group and lower than that in the WT group. All values are expressed as the mean ± SD (*p < 0.05, **p < 0.01, compared with the AD group).

These aspects are well represented in the pharmacokinetic (PK) parameters (Table 2). In the AD group, all area under the curve (AUC) values of the target regions were 27–38% lower than the corresponding values in WT mice (Fig. 2F–J). The taurine group showed a 37–46% higher AUC value than the AD group. The maximum concentration (C_max_) values of the target regions occurred between 1.95 and 2.48 min in the WT group, and the AD group exhibited comparable C_max_ values (1.67–2.44). However, the C_max_ values of the AD_Taurine_ group increased compared to those of the other groups (2.41–3.30). According to the results of the peak arrival times (T_max_), ^18^F-FPEB reached a maximum concentration at 3.25 min p.i in the WT group. However, the T_max_ values in the AD and AD_Taurine_ groups were shorter than that in the WT group (AD: 1.55–2.34 min, AD_Taurine_: 1.35–1.45 min).

**Table 2:** Comparison of PK parameters for ^18^F-FPEB. The C_max_ values of the AD_Taurine_ group increased compared to those of the other groups.

Group	Regions	Pharmacokinetic parameters		
		C_max_ (SUV)	T_max_ (min)	AUC (SUV x min)
WT	Cortex	1.95 ± 0.56	3.25 ± 0.01	80.43 ± 7.88
	Striatum	2.42 ± 0.66	3.35 ± 0.22	106.29 ± 15.68
	Hippocampus	2.48 ± 0.65	3.25 ± 0.01	106.65 ± 11.68
	Thalamus	2.47 ± 0.69	3.25 ± 0.01	59.06 ± 8.55
AD	Cortex	1.67 ± 0.16	1.55 ± 0.45	53.60 ± 3.76
	Striatum	2.34 ± 0.15	2.34 ± 0.15	70.79 ± 9.71
	Hippocampus	2.30 ± 0.23	1.65 ± 0.42	70.16 ± 9.32
	Thalamus	2.44 ± 0.29	1.45 ± 0.45	45.87 ± 6.30
AD_Taurine_	Cortex	2.41 ± 0.18***	1.45 ± 0.27	74.02 ± 11.54**
	Striatum	3.12 ± 1.42	1.45 ± 0.27	93.62 ± 11.05**
	Hippocampus	3.04 ± 1.39	1.35 ± 0.22	93.35 ± 13.72*
	Thalamus	30.30 ± 1.54	1.35 ± 0.22	59.20 ± 11.38*

All values are expressed as the mean ± SD (*p < 0.05, **p < 0.01, ***p < 0.001 compared with the AD group). All statistical analysis was performed using Prism software (version 8).

### Distribution volume ratios (DVRs)

To elucidate the specific binding level for metabolic glutamate receptor type 5 (mGluR5), we calculated regional DVR values (Table 3). The mean DVR values for the AD group were 29–42% lower than those for the WT group. However, the AD_Taurine_ group showed 15–20% higher DVR values than the AD group.

**Table 3:** Comparison of regional DVR values. The binding values in the AD_Taurine_ group were significantly higher than those in the AD group.

Group	Distribution volume ratios			
	Cortex	Striatum	Hippocampus	Thalamus
WT	3.84 ± 0.25	4.78 ± 0.33	4.96 ± 0.25	2.45 ± 0.05
AD	2.41 ± 0.14	2.89 ± 0.31	2.89 ± 0.25	1.73 ± 0.14
AD_Taurine_	2.80 ± 0.18**	3.45 ± 0.28**	3.46 ± 0.38**	1.99 ± 0.18*

Data are presented as the mean ± SD. Statistical significance was defined as a p value less than 0.05 for comparisons between groups. (*p < 0.05, **p < 0.01 compared with the AD group). All statistical analysis was performed using Prism software (version 8).

### Immunoblotting

**Figure 3:**
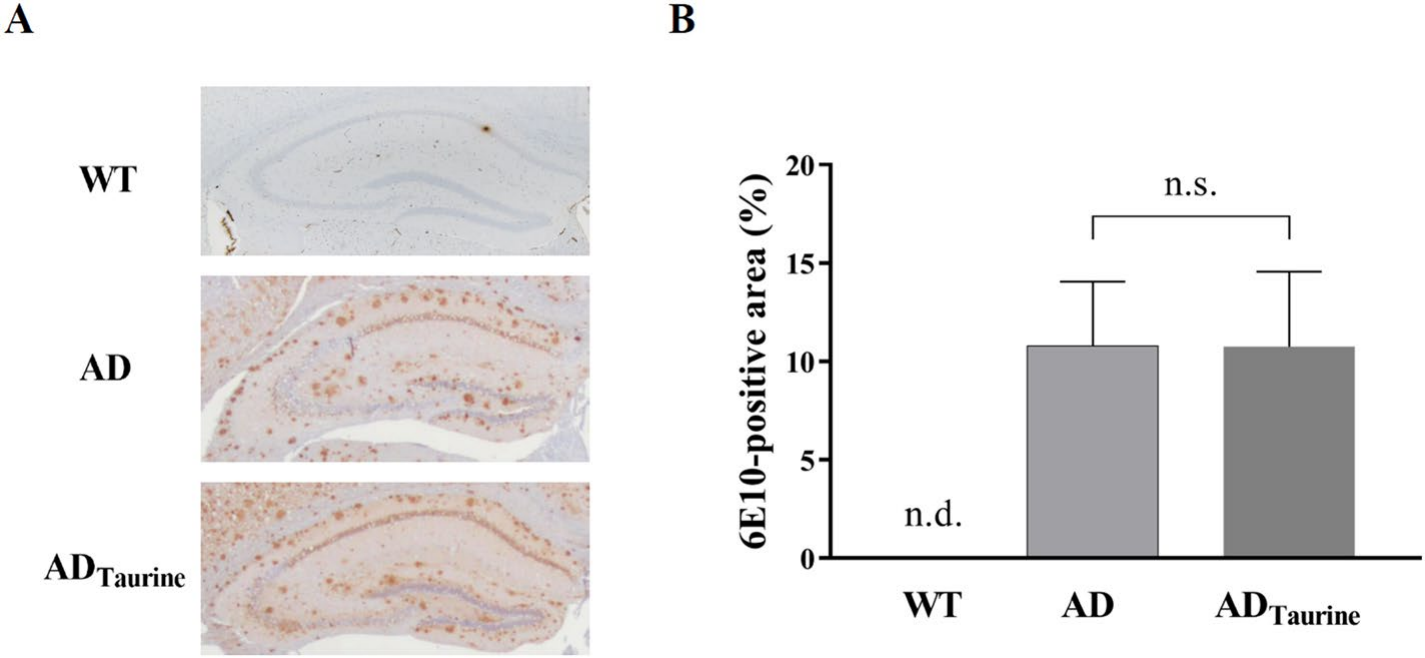
Immunohistochemical staining of Aβ in the brains of AD and AD_Taurine_ mice (**A**). The insets represent high-magnification images of the hippocampus. No morphological difference in Aβ was observed between the AD and AD_Taurine_ groups. Quantification of Aβ deposition in the hippocampus (**B**). No quantitative differences were observed between the AD and AD_Taurine_ groups. Values are presented as the mean ± SD, n.s. = statistically nonsignificant difference, n.d. = not detected. All statistical analysis was performed using Prism software (version 8).

To determine whether the actual Aβ burden was affected by taurine, we performed immunohistochemistry (Fig. 3). As shown in Fig. 3A, Aβ deposition was not detected only in the WT mice. No morphological differences in Aβ were observed between the AD and AD_Taurine_ groups. Additionally, no difference in Aβ deposition was observed between the two groups in the quantitative analysis (Fig. 3B, AD: 10.8 ± 2.8 vs AD_Taurine_: 10.8 ± 3.3, *p* > 0.05).

## Discussion

Early intervention for AD is known to have a greater positive effect than interventions during middle or late stages. Amyloid plaque deposition begins in mice of the 5xFAD strain at 2 months of age^25^. Therefore, in this study, taurine supplementation was started at 2 months of age to determine the therapeutic potential of taurine in the early stages of AD. Although taurine supplementation did not reduce amyloid pathology in the AD animal model, it significantly increased brain uptake of mGluR5. PK analysis also showed that the maximum concentration values were elevated by taurine, indicating that taurine may increase cerebral blood flow. Therefore, early treatment with taurine facilitated recovery of the glutamate system in AD. This is the first study to evaluate the therapeutic effect of taurine in AD using molecular imaging.

Many studies have reported that the glutamate system is associated with the progression of AD^26–28^. Caroline et al. reported that deregulation of glutamate-mediated excitatory signaling is a common mechanism in AD^29^. Renner et al.^30^ reported that the Aβ oligomer (AβO) directly causes deleterious effects in glutamate neurotransmission, leading to elevated intracellular calcium levels. In addition, Hamilton et al.^31^ showed that pathological glutamate signaling contributes to neuronal cell death. Glutamate receptor antagonists have been developed to treat AD, and their therapeutic efficiency has been verified. However, side effects, such as nausea, anorexia, dizziness and headache, which decrease the quality of life of patients, have been reported^6, 7^.

Taurine has not been reported to cause specific adverse events in clinical or preclinical cases. Previous clinical studies have reported that taurine does not induce genotoxic, carcinogenic or teratogenic effects in stroke or heart ischemia patients^32–34^. In Murakami et al.’s study^35^, no specific side effects were observed when C57BL/6J mice were treated with taurine for 6 months. Our study also revealed no severe side effects in rodents despite the long-term use of taurine for 7 months. We did not observe any changes in hair loss, water consumption or body weight in the mice throughout the experiment. In addition, no specific macroscopic changes were found upon autopsy. These results suggest that taurine has the potential to treat AD without side effects.

Biologically, taurine plays several crucial roles in the modulation of calcium signaling, osmoregulation, and membrane stabilization^36, 37^. The sulfonic acid group in taurine has been reported to bind to Aβ and prevent glycosaminoglycans (GAGs) from binding to Aβ. In AD patients, amyloid peptide binds to GAGs, causing plaques to accumulate in the brain and destroy neurons^38^. Another clinical study reported that 3-amino-1-propane sulfonic acid (3-APS) was designed as an anti-amyloid therapy and significantly reduces Aβ in the brain^39^. Taurine has structural similarity to 3-APS; therefore, we postulated that taurine would eventually bind Aβ directly to inhibit the interaction of GAGs with amyloid peptide^20^. This series of events is thought to eventually led to downregulation of mGluR5. Many studies in rodents also have demonstrated the effects of taurine on AD. Kim et al.^40^ reported that taurine significantly ameliorated hippocampus-related cognitive deficits in an AD mouse model. In another recent study, taurine was reported to bind directly to AβO and consequently ameliorated the behavioral deficiencies of AD, such as the loss of learning and memory^18^. Roberto et al.^26^ reported that taurine strongly protected neurons against the neurotoxicity of Aβ in vitro. Thy also demonstrated that taurine prevented neurotoxicity caused by Aβ and glutamate receptor agonists in an in vitro study. However, the studies referenced above were all performed based on ex vivo or behavioral observations rather than changes at the molecular level. The progression of AD begins preferentially with a change at the molecular level, and then clinical symptoms appear due to functional and structural changes in the brain. The novelty of the present study is that the therapeutic effects of taurine on AD were evaluated via functional PET.

Although the mechanism by which taurine regulates glutamatergic signaling is not yet clear, taurine may act as a modulator against AD in two manners. First, taurine is expected to play a role in facilitating the regulation of calcium signaling homeostasis in the brain, thereby leading to recovery of the glutamatergic system. Intracellular Ca^2+^ signaling is fundamental to neuronal physiology; therefore, disruptions in Ca^2+^ homeostasis are implicated in neuronal diseases, including AD^41^. Under physiological conditions, when calcium levels are increased in the mitochondria, Ca^2+^ is removed by a sodium-calcium exchanger. Such feedback helps maintain homeostasis of cellular calcium levels^42^. However, under pathological conditions, Aβ accumulation leads to upregulation of intracellular Ca^2+^ in mitochondria, which induces neuronal death^43^. Extensive evidence indicates that Aβ causes dysregulation of calcium signaling. Kim et al.^44^ reported that Aβ_1–42_ causes mitochondrial depolarization and increases dysregulated cellular Ca^2+^ levels. Weiss et al.^45^ showed that nimodipine, a Ca^2+^ channel blocker, reduced Aβ levels, implying that calcium homeostatic mechanisms are involved in Aβ neurotoxicity. Jakaria et al.^46^ also suggested that taurine reduced abnormal Ca^2+^ signaling in sensory neurons, which reduced glutamate-mediated toxicity. Second, taurine regulates AβO accumulation, eventually leading to upregulated glutamate. Previous studies showed that taurine inhibited the accumulation of amyloid plaques and prevented neurotoxicity in AD^20, 26^. However, in our immunohistochemistry analysis, no difference in Aβ plaque concentrations was found between the taurine-treated group and the AD group. The reason for this result is not obvious, but we hypothesize that taurine may be involved in a mechanism inhibiting the toxicity of AβO rather than Aβ plaques. Soluble AβO appears to be a more toxic and disease-relevant element in AD pathogenesis than plaques^47–49^. Jang et al.^18^ reported that taurine did not affect Aβ plaque levels in the APP/PS1 model but interacted directly with AβO, which led to enhanced memory function. Lesné et al.^50^ reported that Aβ plaques did not induce memory impairment in the absence of AβO in Tg2576 mice. Gandy et al.^51^ revealed that soluble AβO induced impaired cognitive function in an AD mouse model without Aβ plaques. Further investigation is required to address this issue.

The present study had some limitations. First, we did not perform glutamatergic signal measurements using nanomaterial-based biosensors. The level of glutamate was assessed only by PET images, and the exact molecular mechanism of taurine in AD cannot be determined from such images. Second, the number of animals included in the imaging and histological analyses was too few to derive solid support for the imaging findings (n = 5 for each group). Third, the most appropriate therapeutic intervention time could not be concluded from this study. We began treating AD mice with taurine at 2 months of age, representing an early therapeutic intervention. Therefore, the effects of administering taurine to aged mice remain unknown. Fourth, biochemical information supporting the effect of taurine on improving glutamatergic signaling is lacking, which needs to be addressed in a future study. Last, we did not include taurine-treated WT mice as a positive control group, which may have more clearly revealed the AD-specific treatment effects of taurine. In summary, although taurine treatment did not completely recover the glutamatergic system, it caused increased brain uptake of mGluR5 on PET and specific binding in the AD animal model. According to these results, taurine exerts a potential therapeutic effect in AD.

## Methods

### Animals

The care, maintenance, and treatment of animals in these studies followed protocols approved by the Institutional Animal Care and Use Committee of Korea Institute of Radiological & Medical Sciences (KIRAMS), and the experiments involving animals were performed according to the Guide for the Care and Use of Laboratory Animals published by the US National Institutes of Health. The animal housing chambers were automatically controlled at a temperature of 22 ± 3 °C and 55 ± 20% humidity under a 12-h light/dark cycle. A sterilized rodent diet and purified tap water were supplied ad libitum.

### Drugs

Taurine was purchased from Sigma-Aldrich (St. Louis, Missouri, USA).

### Study protocol

**Figure 4:**
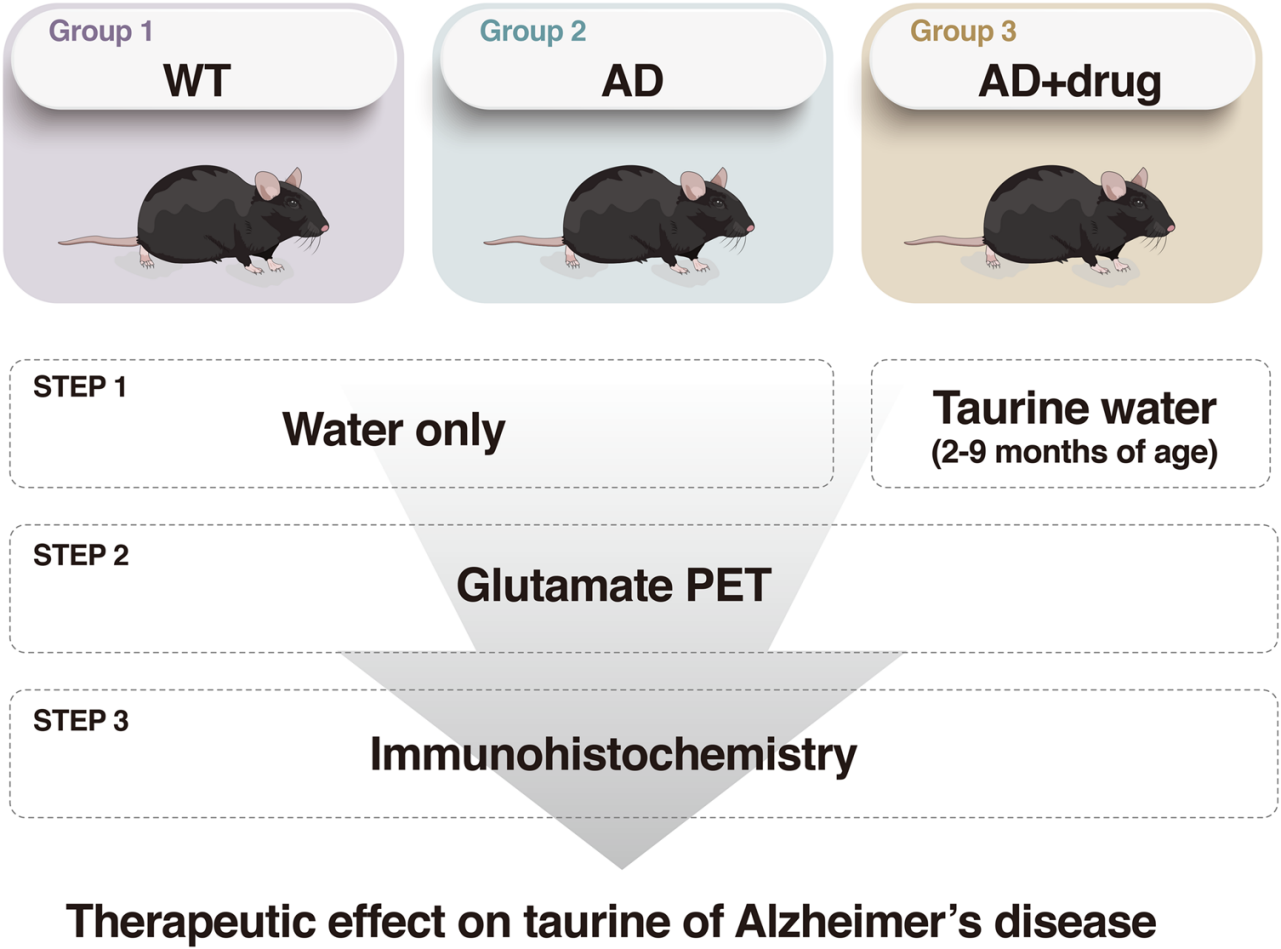
Schematic of the study process.

Three groups of mice were used for these studies: B6/SJL F1 hybrids (wild-type (WT), n = 5), 5xFAD mice (AD, n = 5), and 5xFAD mice treated with taurine by oral administration in drinking water from 2 to 9 months of age (AD_Taurine_, n = 5). Taurine was administered at a dose of 1000 mg/kg/day, which was reported to correspond to reduced hippocampal Aβ and behavioral improvements in AD mice in a previous study^40^. To calculate the taurine dose, the amount of water intake per mouse was calculated by measuring water consumption every week in each cage. The weight of each animal was also measured weekly to calculate the taurine dose. At 9 months of age, all groups underwent glutamate PET. After the imaging study was completed, the animals were sacrificed, and brain tissue samples were prepared. Immunohistochemistry experiments were performed to quantify mGluR5 in the target regions. A detailed study protocol is illustrated in Fig. 4.

### Preparation of ^18^F-FPEB

^18^F-FPEB was synthesized by nucleophilic substitution of F-18 on the precursor. The tracer was prepared according to a previously described procedure^52^. The mean radiochemical purity was 99%.

### PET/CT scan

PET images of the mice were obtained using a small animal PET scanner (NanoScan, Mediso, Budapest, Hungary). The scanner has a peak absolute system sensitivity of > 9% in the 250–750 keV energy window, an axial field of view of 28 cm, a trans axial field of view of 35–120 mm and a trans axial resolution of 0.7 mm at 1 cm off center. Mice were anesthetized with 2.0% isoflurane, and ^18^F-FPEB (9.4 ± 1.2 MBq/200 µL) was injected through the tail vein with a syringe pump (KDS 210, KD scientific, Holliston, MA) for 1 min. Simultaneously, a dynamic PET scan was performed for 60 min, and images were reconstructed using the 3-dimensional ordered subset expectation maximization (3D-OSEM) algorithm with 4 iterations (14 × 30 s, 3 × 60 s, 4 × 300 s, 3 × 600 s, 24 frames in total). The imaging scans were acquired with an energy window of 400–600 keV. All images were reconstructed using the 3D-OSEM algorithm (4 iterations, 6 subsets). For attenuation correction and anatomical information, computed tomography (CT) scans were acquired immediately after PET (50 kVp of X-ray voltage with 0.16 mAs and a 520-µA anode current).

### Image analysis

**Figure 5:**
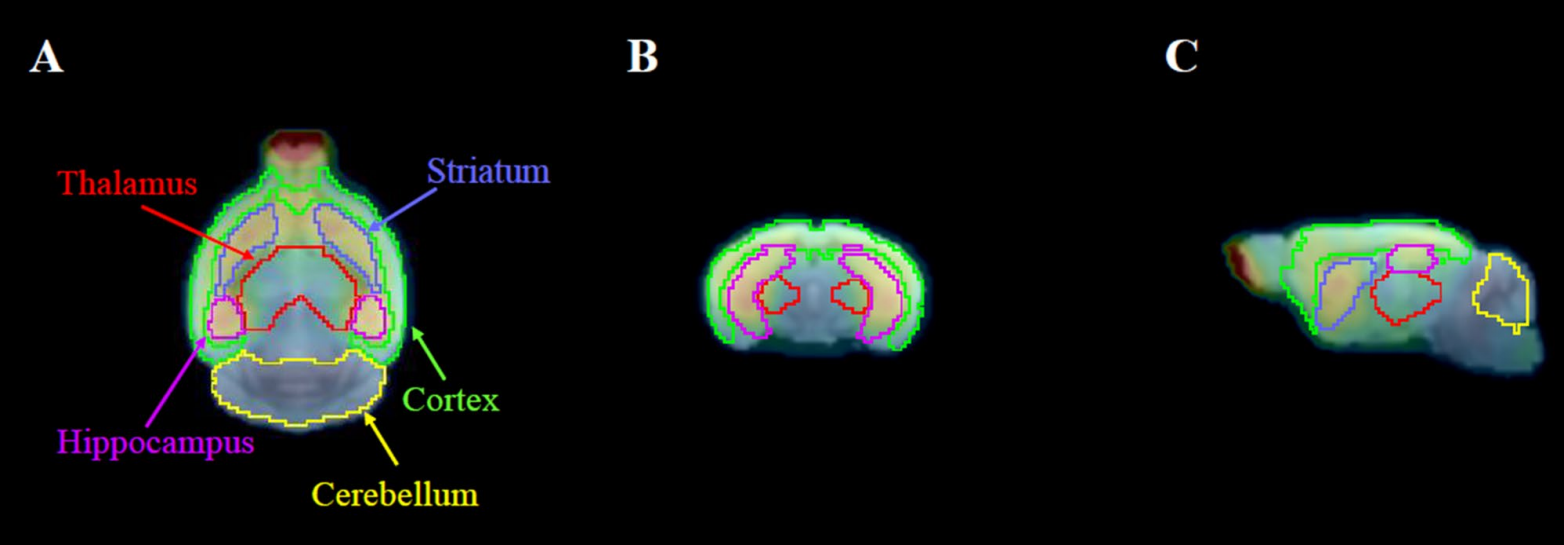
Definition of VOIs in an AD mouse in the horizontal (**A**), coronal (**B**), and sagittal (**C**) planes. VOI applied PET images were produced using the PMOD fusion tool (version 3.4).

For the analysis of mouse brain data, a house-made brain MR template for 5xFAD was used^53^. For motion correction, all dynamic PET images were realigned to the mean PET images (0–24 frames) by the sum of the squared difference dissimilarity measure and the Powell algorithm (PMOD 3.4, PMOD Technologies Ltd, Switzerland). Considering that transient equilibrium for ^18^F-FPEB was reached after an average of 30 min, we used the mean PET images from the time windows of 30–60 min in dynamic PET. Then, each mean PET image was spatially normalized to the T2-weighted mouse brain MR template (M. Mirrione), which is embedded in PMOD software. Finally, individual dynamic PET images for all groups were spatially normalized to the MR template, masked to exclude extracerebral signals and smoothed with a 3D Gaussian filter (FWHM = 1.0 mm). The cortex, striatum, hippocampus, thalamus and cerebellum were selected as volumes of interest (VOIs) on the MR template (Fig. 5). Decay-corrected regional time-activity curves (TACs) were acquired from VOIs and normalized to account for differences in injected doses and body weights to yield units of the standardized uptake value (SUV). The SUV values obtained for each region of activity were multiplied by the body weight divided by the injected dose for each animal. PK parameters were estimated from TACs using Prism software (version 8, GraphPad Software, Inc., USA). AUC values were obtained from 10 to 60 min by the trapezoid rule. The T_max_ and the C_max_ were also compared between groups. In addition, the DVR was estimated using Logan graphical analysis to evaluate receptor binding density^54^. Using the Simplified Reference Tissue Model (SRTM), the individual clearance rate (k2′) was obtained from the TACs of target regions (t∗ = 10 min) and then applied to each DVR calculation.

### Immunohistochemistry

After PET imaging studies, immunostaining experiments were performed. The test was performed as previously described^25, 53^. Two age-matched mice per group were sacrificed, and then their brains were extracted. Formalin-fixed mouse brains were first cranially divided into the hippocampal region (from − 1.94 to − 1.58 mm at the bregma) using disposable blades, embedded into paraffin and sectioned at 5-µm intervals. Immunohistochemistry was conducted using a Vectastain Elite ABC kit (Vector Laboratories Inc., Burlingame, CA, USA) following the manufacturer’s protocol. For antigen retrieval, the sections were placed in citrate buffer (pH 6.0) and heated in boiling water for 30 min. The sections were then placed in 0.3% H_2_O_2_ in absolute methanol for 15 min at room temperature to block endogenous peroxidase. The sections were incubated overnight at 4 °C with mouse anti-6E10 antibody (1:1000, SIG-39320, Covance, Emeryville, CA), washed and incubated with the corresponding secondary antibody. ImageJ was used to quantify the amount of Aβ in the hippocampus. As a control, the primary antibody was omitted from several test sections in each experiment. The sections were counterstained with Harris’ hematoxylin prior to mounting.

### Statistical analysis

The quantitative results are expressed as the means ± SD. All statistical results were analyzed with Prism software. Student’s t-test was used to determine statistical significance at the 95% confidence level, and *p* < 0.05 was considered significantly different.
